# Supplementary material for: Transcriptomic Analysis of the Dehydration Rate of Mature Rice (Oryza sativa) Seeds
Source: Int J Mol Sci. 2023 Jul 16;24(14):11527. doi: 10.3390/ijms241411527 (PMC10380403; doi:10.3390/ijms241411527)
Supplement: Supplementary file 1 [file ijms-24-11527-s001.zip › Table S 1-8í¬ijms.pdf]

Table S 1 Results of RNA quality in twelve experimental seeds

Sample Name	Density (ng/ μL)	OD260/ 280	OD260/ 230	28S/ 18S	RIN
CNSF5-1	960	2.07	1.15	1.90	9.60
CNSF5-2	610	1.88	1.81	1.80	8.30
CNSF5-3	1320	2.06	1.19	1.80	8.70
NSF5-1	1395	2.11	1.56	1.50	7.60
NSF5-2	1010	2.19	1.48	1.60	7.70
NSF5-3	875	1.90	1.55	1.70	8.00
CNSF75-1	426	1.99	0.57	1.80	8.70
CNSF75-2	780	2.01	1.03	1.90	9.00
CNSF75-3	930	1.94	1.59	1.90	8.90
NSF75-1	735	2.04	0.87	1.50	8.20
NSF75-2	418	2.01	0.82	2.20	8.80
NSF75-3	585	2.06	1.00	1.50	8.20

Table S 2 Statistical table of seed transcriptome sequencing quality of twelve dehydrated and dried samples

sample_name	Total raw read（M）	Total clean read（M）	Total clean base（Gb）	Clean read Q20（%）	Clean read Q30（%）	GC (%)
CNSF5-1	23.92	23.76	1.19	98.24	94.89	51.99
CNSF5-2	23.92	23.79	1.19	98.23	94.85	51.12
CNSF5-3	23.92	23.70	1.19	97.98	94.24	52.19
NSF5-1	23.92	23.75	1.19	98.12	94.55	52.98
NSF5-2	23.92	23.74	1.19	97.88	93.92	52.70
NSF5-3	23.92	23.57	1.18	98.17	94.65	52.15
CNSF75-1	23.92	23.66	1.18	97.85	93.77	52.14
CNSF75-2	23.92	23.77	1.19	97.84	93.73	51.58
CNSF75-3	23.92	23.81	1.19	98.29	94.76	51.30
NSF75-1	23.92	23.84	1.19	98.24	94.63	52.21
NSF75-2	23.92	23.83	1.19	98.17	94.32	51.67
NSF75-3	23.92	23.79	1.19	98.30	94.76	51.41

Table S 3 Comparative analysis of the transcriptome of seeds from twelve dehydrated samples and the genome of Charybdis Japonica

Sample name	Total clean read（M）	Total Mapping(%)	Uniquely Mapping(%)
CNSF5-1	23.76	91.90	79.55
CNSF5-2	23.79	92.43	79.31
CNSF5-3	23.70	92.95	80.67
NSF5-1	23.75	93.15	83.75
NSF5-2	23.74	92.65	82.28
NSF5-3	23.57	93.18	80.60
CNSF75-1	23.66	94.82	82.75
CNSF75-2	23.77	95.36	82.94
CNSF75-3	23.81	95.00	81.96
NSF75-1	23.84	95.01	83.49
NSF75-2	23.83	95.06	83.59
NSF75-3	23.79	95.39	82.33

Table S 4：CNSF5 vs NSF5 GO enrichment-related genes

Gene ID	Gene bank annotation	GO Cellular componen	GO Molecular function	GO Biological process	Targeted Protein
LOC107275269	protein argonaute 12		GO:0003676 nucleic acid binding		PF02171:Piwi domain;PF16487:Mid domain of argonaute
LOC107275576	chaperone protein dnaJ A7A, chloroplastic-like	GO:0005737 cytoplasm;GO:0009507 chloroplast;GO:0009535 chloroplast thylakoid membrane;GO:0009536 plastid	GO:0005524 ATP binding;GO:0031072 heat shock protein binding;GO:0046872 metal ion binding;GO:0051082 unfolded protein binding	GO:0006457 protein folding;GO:0009408 response to heat;GO:0042026 protein refolding;GO:0051085 chaperone cofactor-dependent protein refolding	PF00226:DnaJ domain;PF00684:DnaJ central domain;PF01556:DnaJ C terminal domain
LOC107276572	glucose-6-phosphate/phosphate translocator 2, chloroplastic	GO:0005794 Golgi apparatus;GO:0016020 membrane;GO:0016021 integral component of membrane	GO:0015120 phosphoglycerate transmembrane transporter activity;GO:0015297 antiporter activity;GO:0022857 transmembrane transporter activity;GO:0071917 triose-phosphate transmembrane transporter activity	GO:0055085 transmembrane transport	PF03151:Triose-phosphate Transporter family
LOC107276946	uncharacterized LOC107276946				
LOC107278284	uncharacterized LOC107278284				PF10714:Late embryogenesis abundant protein 18

Continued Table S 4

Gene ID	Gene bank annotation	GO Cellular componen	GO Molecular function	GO Biological process	Targeted Protein
LOC107278869	uncharacterized LOC107278869				
LOC107278890	uncharacterized LOC107278890				
LOC107279351	protein MAIN-LIKE 1-like				
LOC107279571	uncharacterized LOC107279571				
LOC107279585	ATP synthase subunit 9, mitochondrial				
LOC107279587	uncharacterized LOC107279587				
LOC107279602	uncharacterized LOC107279602				
LOC112936894	uncharacterized LOC112936894				
LOC112937002	ATP synthase subunit a-like	GO:0005743 mitochondrial inner membrane;GO:0016021 integral component of membrane;GO:0045263 proton-transporting ATP synthase complex, coupling factor F(o)	GO:0015078 proton transmembrane transporter activity;GO:0016787 hydrolase activity	GO:0015986 ATP synthesis coupled proton transport	
LOC112937433	NADH dehydrogenase [ubiquinone] iron-sulfur protein 2				
LOC112938716	uncharacterized LOC112938716				

Continued Table S 4

Gene ID	Gene bank annotation	GO Cellular componen	GO Molecular function	GO Biological process	Targeted Protein
LOC112939055	disease resistance protein TAO1-like				
LOC4325331	uncharacterized LOC4325331				PF14649:Spatacsin C-terminus
LOC4325373	protein trichome birefringence-like 38	GO:0005794 Golgi apparatus	GO:0016413 O-acetyltransferase activity		PF13839:GDSL/SGNH-like Acyl-Esterase family found in Pmr5 and Cas1p;PF14416:PMR5 N terminal Domain
LOC4325694	16.6 kDa heat shock protein-like	GO:0005737 cytoplasm	GO:0043621 protein self-association;GO:0051082 unfolded protein binding	GO:0000302 response to reactive oxygen species;GO:0006457 protein folding;GO:0009408 response to heat;GO:0009651 response to salt stress;GO:0042542 response to hydrogen peroxide;GO:0051259 protein complex oligomerization	PF00011:Hsp20/alpha crystallin family
LOC4325848	uncharacterized LOC4325848	GO:0005623 cell;GO:0043291 RAVE complex		GO:0007035 vacuolar acidification	PF00400:WD domain, G-beta repeat;PF12234: RAVE protein 1 C terminal

Continued Table S 4

Gene ID	Gene bank annotation	GO Cellular componen	GO Molecular function	GO Biological process	Targeted Protein
LOC4327816	E3 ubiquitin-protein ligase listerin	GO:0005829 cytosol;GO:1990112 RQC complex	GO:0008270 zinc ion binding;GO:0043023 ribosomal large subunit binding;GO:0061630 ubiquitin protein ligase activity	GO:0043161 proteasome-mediated ubiquitin-dependent protein catabolic process;GO:0072344 rescue of stalled ribosome;GO:1990116 ribosome-associated ubiquitin-dependent protein catabolic process	PF13639:Ring finger domain
LOC4327988	thiosulfate sulfurtransferase 18				PF00581:Rhodanese-like domain
LOC4330777	protein EXORDIUM				PF04674:Phosphate-induced protein 1 conserved region
LOC4332360	17.4 kDa class I heat shock protein-like	GO:0005737 cytoplasm	GO:0043621 protein self association; GO:0051082 unfolded protein binding	GO:0000302 response to reactive oxygen species;GO:0006457 protein folding;GO:0009408 response to heat;GO:0009651 response to salt stress;GO:0042542 response to hydrogen peroxide; GO:0045471 response to ethanol;GO:0046685 response to arsenic-containing substance; GO:0046686 response to cadmium ion;GO:0046688 response to copper ion; GO:0051259 protein complex oligomerization	PF00011:Hsp20/ alpha crystallin family

Continued Table S 4

Gene ID	Gene bank annotation	GO Cellular componen	GO Molecular function	GO Biological process	Targeted Protein
LOC4332361	18.1 kDa class I heat shock protein-like	GO:0005737 cytoplasm	GO:0043621 protein self-association;GO:0051082 unfolded protein binding	GO:0000302 response to reactive oxygen species;GO:0006457 protein folding;GO:0009408 response to heat;GO:0009651 response to salt stress;GO:0042542 response to hydrogen peroxide; GO:0045471 response to ethanol;GO:0046685 response to arsenic-containing substance; GO:0046686 response to cadmium ion;GO:0046688 response to copper ion;GO:0051259 protein complex oligomerization	PF00011:Hsp20/ alpha crystallin family
LOC4332363	17.7 kDa class I heat shock protein-like	GO:0005737 cytoplasm	GO:0043621 protein self-association;GO:0051082 unfolded protein binding	GO:0000302 response to reactive oxygen species;GO:0006457 protein folding;GO:0009408 response to heat;GO:0009651 response to salt stress;GO:0042542 response to hydrogen peroxide;GO:0045471 response to ethanol;GO:0046685 response to arsenic-containing substance;GO:0046686 response to cadmium ion;GO:0046688 response to copper ion;GO:0051259 protein complex oligomerization	PF00011:Hsp20/ alpha crystallin family
LOC4333036	uncharacterized LOC4333036				
LOC4333193	3-oxo-Delta(4,5)-steroid 5-beta-reductase				

Continued Table S 4

Gene ID	Gene bank annotation	GO Cellular componen	GO Molecular function	GO Biological process	Targeted Protein
LOC4333199	probable esterase D14L	GO:0005634 nucleus;GO:0005737 cytoplasm	GO:0016787 hydrolase activity	GO:0036377 arbuscular mycorrhizal association;GO:0080167 response to karrikin	PF12697;Alpha/ beta hydrolase family
LOC4333877	heat shock 70 kDa protein BIP2	GO:0005634 nucleus;GO:0005737 cytoplasm;GO:0005783 endoplasmic reticulum;GO:0005788 endoplasmic reticulum lumen;GO:0016020 membrane;GO:0031410 cytoplasmic vesicle;GO:0034663 endoplasmic reticulum chaperone complex	GO:0000166 nucleotide binding;GO:0005524 ATP binding;GO:0016887 ATPase activity;GO:0031072 heat shock protein binding;GO:0042623 ATPase activity, coupled;GO:0044183 protein binding involved in protein folding;GO:0051082 unfolded protein binding;GO:0051787 misfolded protein binding	GO:0006986 response to unfolded protein;GO:0030433 ubiquitin-dependent ERAD pathway;GO:0030968 endoplasmic reticulum unfolded protein response;GO:0034620 cellular response to unfolded protein;GO:0042026 protein refolding;GO:0051085 chaperone cofactor-dependent protein refolding	PF00012;Hsp70 protein
LOC4334045	serine/threonine-protein kinase SMG1				PF02260;FATC domain

Continued Table S 4

Gene ID	Gene bank annotation	GO Cellular componen	GO Molecular function	GO Biological process	Targeted Protein
LOC4334080	heat stress transcription factor A-2a-like	GO:0005634 nucleus;GO:0005737 cytoplasm;GO:0042025 host cell nucleus	GO:0000978 RNA polymerase II proximal promoter sequence-specific DNA binding;GO:0003677 DNA binding;GO:0003700 DNA-binding transcription factor activity;GO:0043565 sequence-specific DNA binding	GO:0006355 regulation of transcription, DNA-templated;GO:0034605 cellular response to heat;GO:0043618 regulation of transcription from RNA polymerase II promoter in response to stress;GO:0061408 positive regulation of transcription from RNA polymerase II promoter in response to heat stress	PF00447:HSF-type DNA-binding
LOC4335189	uncharacterized LOC4335189				
LOC4337864	pyrophosphate-energized vacuolar membrane proton pump 1	GO:0016021 integral component of membrane	GO:0004427 inorganic diphosphatase activity;GO:0009678 hydrogen-translocating pyrophosphatase activity		PF03030:Inorganic H+ pyrophosphatase
LOC4337900	pre-mRNA-processing-splicing factor 8A	GO:0005681 spliceosomal complex		GO:0000398 mRNA splicing, via spliceosome	PF08082:PRO8NT (NUC069), PrP8 N-terminal domain;PF08083:PROCNSF (NUC071) domain

Continued Table S 4

Gene ID	Gene bank annotation	GO Cellular componen	GO Molecular function	GO Biological process	Targeted Protein
LOC4338718	chitinase 2-like	GO:0031410 cytoplasmic vesicle	GO:0004568 chitinase activity;GO:0008061 chitin binding;GO:0008843 endochitinase activity	GO:0000272 polysaccharide catabolic process;GO:0005975 carbohydrate metabolic process;GO:0006032 chitin catabolic process;GO:0016998 cell wall macromolecule catabolic process;GO:0050832 defense response to fungus;GO:0051707 response to other organism	PF00182:Chitinase class I; PF00187:Chitin recognition protein
LOC4339670	ethylene-responsive transcription factor ERF061	GO:0005634 nucleus	GO:0003677 DNA binding;GO:0003700 DNA-binding transcription factor activity	GO:0010200 response to chitin	PF00847;AP2 domain
LOC4341225	cationic amino acid transporter 8, vacuolar	GO:0005774 vacuolar membrane;GO:0005886 plasma membrane	GO:0022857 transmembrane transporter activity		PF13520:Amino acid permease; PF13906:C-terminus of AA_permease
LOC4341326	putative heat stress transcription factor A-6a	GO:0005634 nucleus;GO:0005737 cytoplasm;GO:0042025 host cell nucleus	GO:0000978 RNA polymerase II proximal promoter sequence-specific DNA binding;GO:0003677 DNA binding;GO:0003700 DNA-binding transcription factor activity;GO:0043565 sequence-specific DNA binding	GO:0006355 regulation of transcription, DNA-templated;GO:0034605 cellular response to heat;GO:0043618 regulation of transcription from RNA polymerase II promoter in response to stress;GO:0061408 positive regulation of transcription from RNA polymerase II promoter in response to heat stress	PF00447:HSF-type DNA-binding

Continued Table S 4

Gene ID	Gene bank annotation	GO Cellular componen	GO Molecular function	GO Biological process	Targeted Protein
LOC4342103	serine/threonine-protein kinase ATR-like	GO:0005634 nucleus;GO:0009536 plastid	GO:0004674 protein serine/threonine kinase activity;GO:0005524 ATP binding;GO:0016301 kinase activity	GO:0000077 DNA damage checkpoint;GO:0000723 telomere maintenance;GO:0006281 DNA repair;GO:0007049 cell cycle	PF00454:Phosphatidy linositol 3- and 4-kinase;PF02259:FAT domain; PF02260:FATC domain;PF08064:UME (NUC010) domain
LOC4344618	cysteine-rich repeat secretory protein 55	GO:0031410 cytoplasmic vesicle			PF01657:Salt stress response/ antifungal
LOC4345509	uncharacterized LOC4345509	GO:0005634 nucleus;GO:0005694 chromosome;GO:0005739 mitochondrion	GO:0008168 methyltransferase activity;GO:0008270 zinc ion binding;GO:0016740 transferase activity;GO:0018024 histone-lysine N-methyltransferase activity	GO:0016571 histone methylation;GO:0032259 methylation;GO:0034968 histone lysine methylation	PF00856:SET domain;PF02182:SAD/SRA domain; PF05033:Pre-SET motif
LOC4348651	sulfate transporter 3.1	GO:0005887 integral component of plasma membrane;GO:0016020 membrane;GO:0016021 integral component of membrane	GO:0008271 secondary active sulfate transmembrane transporter activity;GO:0015301 anion:anion antiporter activity		PF00916:Sulfate permease family; PF01740:STAS domain
LOC4349819	ABC transporter C family member 10	GO:0016020 membrane;GO:0016021 integral component of membrane;GO:0031410 cytoplasmic vesicle			

Continued Table S 4

Gene ID	Gene bank annotation	GO Cellular componen	GO Molecular function	GO Biological process	Targeted Protein
LOC4350805	serine/arginine repetitive matrix protein 1			GO:0051513 regulation of monopolar cell growth	PF14383:DUF761-associated sequence motif
LOC9266741	uncharacterized LOC9266741				PF04862:Protein of unknown function (DUF642)
LOC9267065	chromatin structure-remodeling complex protein SYD		GO:0005524 ATP binding;GO:0042393 histone binding		
LOC9267323	uncharacterized LOC9267323				PF07762:Protein of unknown function (DUF1618)
LOC9267997	heat shock protein 82	GO:0005737 cytoplasm;GO:0005829 cytosol;GO:0005886 plasma membrane;GO:0009986 cell surface;GO:0032991 protein-containing complex;GO:0048471 perinuclear region of cytoplasm	GO:0005524 ATP binding;GO:0051082 unfolded protein binding	GO:0006457 protein folding;GO:0034605 cellular response to heat;GO:0050821 protein stabilization	PF00183:Hsp90 protein;PF02518:Histidine kinase-, DNA gyrase B-, and HSP90-like ATPase
LOC9268035	leaf-specific thionin	GO:0016021 integral component of membrane		GO:0006952 defense response	
LOC9268124	protein RNA-directed DNA methylation 3-like				

Table S 5：CNSF75 vs NSF75 GO enrichment-related genes

Gene ID	Gene bank annotation	GO Cellular componen	GO Molecular function	GO Biological process	Targeted Protein
LOC107275576	chaperone protein dnaJ A7A, chloroplastic-like	GO:0005737 cytoplasm; GO:0009507 chloroplast; GO:0009535 chloroplast thylakoid membrane; GO:0009536 plastid	GO:0005524 ATP binding; GO:0031072 heat shock protein binding; GO:0046872 metal ion binding;GO:0051082 unfolded protein binding	GO:0006457 protein folding;GO:0009408 response to heat; GO:0042026 protein refolding;GO:0051085 chaperone cofactor-dependent protein refolding	PF00226 DnaJ domain;PF00684 DnaJ central domain;PF01556 DnaJ C terminal domain
LOC107276572	glucose-6-phosphate/phosphate translocator 2, chloroplastic	GO:0005794 Golgi apparatus;GO:0016020 membrane;GO:0016021 integral component of membrane	GO:0015120 phosphoglycerate transmembrane transporter activity;GO:0015297 antiporter activity; GO:0022857 transmembrane transporter activity;GO:0071917 triose-phosphate transmembrane transporter activity	GO:0055085 transmembrane transport	PF03151 Triose-phosphate Transporter family
LOC107279585	ATP synthase subunit 9, mitochondrial				

Continued Table S 5

Gene ID	Gene bank annotation	GO Cellular componen	GO Molecular function	GO Biological process	Targeted Protein
LOC4325696	16.9 kDa class I heat shock protein 3-like	GO:0005737 cytoplasm	GO:0043621 protein self-association;GO:0051082 unfolded protein binding	GO:0000302 response to reactive oxygen species;GO:0006457 protein folding; GO:0009408 response to heat;GO:0009651 response to salt stress;GO:0042542 response to hydrogen peroxide;GO:0051259 protein complex oligomerization	PF00011 Hsp20/alpha crystallin family
LOC4327469	ultraviolet-B receptor UVR8				PF00415 Regulator of chromosome condensation (RCC1) repeat
LOC4329612	ethylene-responsive transcription factor ABR1	GO:0005634 nucleus	GO:0003677 DNA binding;GO:0003700 DNA-binding transcription factor activity		PF00847 AP2 domain

Continued Table S 5

Gene ID	Gene bank annotation	GO Cellular componen	GO Molecular function	GO Biological process	Targeted Protein
LOC4330496	17.8 kDa heat shock protein-like	GO:0005737 cytoplasm	GO:0043621 protein self-association;GO:0051082 unfolded protein binding	GO:0000302 response to reactive oxygen species;GO:0006457 protein folding; GO:0009408 response to heat;GO:0009651 response to salt stress;GO:0042542 response to hydrogen peroxide;GO:0051259 protein complex oligomerization	PF00011 Hsp20/alpha crystallin family
LOC4332360	17.4 kDa class I heat shock protein-like	GO:0005737 cytoplasm	GO:0043621 protein self-association;GO:0051082 unfolded protein binding	GO:0000302 response to reactive oxygen species;GO:0006457 protein folding; GO:0009408 response to heat;GO:0009651 response to salt stress;GO:0042542 response to hydrogen peroxide;GO:0045471 response to ethanol; GO:0046685 response to arsenic-containing substance;GO:0046686 response to cadmium ion;GO:0046688 response to copper ion;GO:0051259 protein complex oligomerization	PF00011 Hsp20/alpha crystallin family

Continued Table S 5

Gene ID	Gene bank annotation	GO Cellular componen	GO Molecular function	GO Biological process	Targeted Protein
LOC4332361	18.1 kDa class I heat shock protein-like	GO:0005737 cytoplasm	GO:0043621 protein self-association;GO:0051082 unfolded protein binding	GO:0000302 response to reactive oxygen species;GO:0006457 protein folding; GO:0009408 response to heat;GO:0009651 response to salt stress;GO:0042542 response to hydrogen peroxide;GO:0045471 response to ethanol; GO:0046685 response to arsenic-containing substance;GO:0046686 response to cadmium ion; GO:0046688 response to copper ion;GO:0051259 protein complex oligomerization	PF00011 Hsp20/alpha crystallin family
LOC4333193	3-oxo-Delta(4,5)-steroid 5-beta-reductase				
LOC4334080	heat stress transcription factor A-2a-like	GO:0005634 nucleus;GO:0005737 cytoplasm;GO:0042025 host cell nucleus	GO:0000978 RNA polymerase II proximal promoter sequence-specific DNA binding;GO:0003677 DNA binding;GO:0003700 DNA-binding transcription factor activity;GO:0043565 sequence-specific DNA binding	GO:0006355 regulation of transcription, DNA-templated;GO:0034605 cellular response to heat; GO:0043618 regulation of transcription from RNA polymerase II promoter in response to stress; GO:0061408 positive regulation of transcription from RNA polymerase II promoter in response to heat stress	PF00447 HSF-type DNA-binding

Continued Table S 5

Gene ID	Gene bank annotation	GO Cellular componen	GO Molecular function	GO Biological process	Targeted Protein
LOC4335556	70 kDa peptidyl-prolyl isomerase	GO:0005737 cytoplasm	GO:0003755 peptidyl-prolyl cis-trans isomerase activity;GO:0016853 isomerase activity	GO:0000413 protein peptidyl-prolyl isomerization;GO:0061077 chaperone-mediated protein folding	PF00254 FKBP-type peptidyl-prolyl cis-trans isomerase;PF13181 Tetratricopeptide repeat
LOC4335956	23.2 kDa heat shock protein-like	GO:0005783 endoplasmic reticulum	GO:0043621 protein self-association;GO:0051082 unfolded protein binding	GO:0000302 response to reactive oxygen species;GO:0006457 protein folding; GO:0009408 response to heat;GO:0009651 response to salt stress;GO:0042542 response to hydrogen peroxide;GO:0051259 protein complex oligomerization	PF00011 Hsp20/ alpha crystallin family
LOC4336524	hsp70-Hsp90 organizing protein		GO:0051879 Hsp90 protein binding		PF00515 Tetratricopeptide repeat;PF07719 Tetratricopeptide repeat;PF13181 Tetratricopeptide repeat;PF13374 Tetratricopeptide repeat;PF13414 TPR repeat; PF17830 STI1 domain

Continued Table S 5

Gene ID	Gene bank annotation	GO Cellular componen	GO Molecular function	GO Biological process	Targeted Protein
LOC4339759	RNA polymerase sigma factor sigE, chloroplastic/mitochondrial	GO:0009536 plastid	GO:0003700 DNA-binding transcription factor activity;GO:0003899 DNA-directed 5'-3' RNA polymerase activity; GO:0016987 sigma factor activity	GO:0006352 DNA-templated transcription, initiation;GO:0006355 regulation of transcription, DNA-templated;GO:2000142 regulation of DNA-templated transcription, initiation	PF04539 Sigma-70 region 3;PF04545 Sigma-70, region 4
LOC4340201	U-box domain-containing protein 70-like		GO:0004842 ubiquitin-protein transferase activity;GO:0016301 kinase activity		PF04564 U-box domain
LOC4340661	16.0 kDa heat shock protein, peroxisomal-like	GO:0005777 peroxisome;GO:0005782 peroxisomal matrix	GO:0043621 protein self-association;GO:0051082 unfolded protein binding	GO:0000302 response to reactive oxygen species;GO:0006457 protein folding; GO:0009408 response to heat;GO:0009651 response to salt stress;GO:0042542 response to hydrogen peroxide;GO:0051259 protein complex oligomerization	PF00011 Hsp20/alpha crystallin family

Continued Table S 5

Gene ID	Gene bank annotation	GO Cellular componen	GO Molecular function	GO Biological process	Targeted Protein
LOC4341326	putative heat stress transcription factor A-6a	GO:0005634 nucleus;GO:0005737 cytoplasm;GO:0042025 host cell nucleus	GO:0000978 RNA polymerase II proximal promoter sequence-specific DNA binding;GO:0003677 DNA binding; GO:0003700 DNA-binding transcription factor activity; GO:0043565 sequence-specific DNA binding	GO:0006355 regulation of transcription, DNA-templated;GO:0034605 cellular response to heat; GO:0043618 regulation of transcription from RNA polymerase II promoter in response to stress; GO:0061408 positive regulation of transcription from RNA polymerase II promoter in response to heat stress	PF00447 HSF-type DNA-binding
LOC4347198	uncharacterized LOC4347198	GO:0016020 membrane;GO:0016021 integral component of membrane			
LOC4347405	heat shock protein 81-3-like	GO:0005737 cytoplasm;GO:0005829 cytosol;GO:0005886 plasma membrane;GO:0009986 cell surface;GO:0032991 protein-containing complex;GO:0048471 perinuclear region of cytoplasm	GO:0000166 nucleotide binding;GO:0005524 ATP binding;GO:0051082 unfolded protein binding	GO:0006457 protein folding;GO:0034605 cellular response to heat;GO:0050821 protein stabilization	PF00183 Hsp90 protein;PF02518 Histidine kinase-, DNA gyrase B-, and HSP90-like ATPase

Continued Table S 5

Gene ID	Gene bank annotation	GO Cellular componen	GO Molecular function	GO Biological process	Targeted Protein
LOC4350180	21.9 kDa heat shock protein-like	GO:0005783 endoplasmic reticulum;GO:0031410 cytoplasmic vesicle	GO:0043621 protein self-association;GO:0051082 unfolded protein binding	GO:0000302 response to reactive oxygen species;GO:0006457 protein folding; GO:0009408 response to heat;GO:0009651 response to salt stress;GO:0042542 response to hydrogen peroxide;GO:0051259 protein complex oligomerization	PF00011 Hsp20/alpha crystallin family
LOC9266706	uncharacterized LOC9266706				
LOC9267997	heat shock protein 82	GO:0005737 cytoplasm;GO:0005829 cytosol;GO:0005886 plasma membrane;GO:0009986 cell surface;GO:0032991 protein-containing complex;	GO:0005524 ATP binding;GO:0051082 unfolded protein binding	GO:0006457 protein folding;GO:0034605 cellular response to heat;GO:0050821 protein stabilization	PF00183 Hsp90 protein;PF02518 Histidine kinase-, DNA gyrase B-, and HSP90-like ATPase
LOC9268751	ethylene-responsive transcription factor ABR1	GO:0005634 nucleus	GO:0003677 DNA binding;GO:0003700 DNA-binding transcription factor activity		

Continued Table S 5

Gene ID	Gene bank annotation	GO Cellular componen	GO Molecular function	GO Biological process	Targeted Protein
LOC9271813	60S ribosomal protein L44	GO:0005840 ribosome;GO:0022625 cytosolic large ribosomal subunit	GO:0003735 structural constituent of ribosome	GO:0006412 translation	PF00935 Ribosomal protein L44

Table S 6：CNSF5 vs NSF5 KEGG Metabolic pathway-related genes

Gene ID	Other Marker No.	Types	Kegg Pathway	Chromosomes	log2 (NSF5 / CNSF5)	log2 (NSF75 / CNSF75)
LOC107275269		mRNA		chr3	-1.139714421	0.169825219
LOC107275576	DJA7/DJA7B/OJ1005_D04.17/OsDjA7	mRNA		chr5	20.6090789	-21.87459296
LOC107276572	OJ1372_D12.152	mRNA		chr7	21.84273896	-23.7561104
LOC107276946		mRNA		chr2	-1.820619284	-0.002876518
LOC107278284		mRNA		chr9	10.78360274	0.163531872
LOC107278869		mRNA		chr9	-1.452441831	-0.069150938
LOC107278890		mRNA		chr9	3.787212218	1.033292993
LOC107279351		mRNA		chr11	-3.536241382	0.511874971
LOC107279571		mRNA		chr12	2.780495558	0.857344067
LOC107279585		mRNA	00190///Oxidative phosphorylation	chr12	1.488743183	1.160579142
LOC107279587		mRNA		chr12	1.599617702	0.833250747
LOC107279602		mRNA		chr12	1.139849663	0.630615963
LOC112936894		mRNA		chr11	-1.028273859	0.245627002
LOC112937002		mRNA		chr1	2.190493425	0.857023105
LOC112937433		mRNA		chr12	1.69388237	0.366729413
LOC112938716		mRNA		chr4	-2.423508169	
LOC112939055		mRNA		chr5	2.276238066	1.567214538
LOC4325331	P0489A01.6	mRNA		chr1	-1.052809499	-0.330538802
LOC4325373		mRNA		chr1	1.081671617	0.013078572
LOC4325694	HSP16.6/OsHsp16.6/OsJ_00276	mRNA	04141///Protein processing in endoplasmic reticulum	chr1	1.50634936	1.377065503
LOC4325848		mRNA		chr1	-1.113465372	-0.494573875
LOC4327816		mRNA		chr1	-1.198814316	-0.373458878
LOC4327988	OJ1435_F07.17	mRNA		chr2	1.718381383	-0.115870859
LOC4330777		mRNA		chr2	1.164665905	-0.532106864

Continued Table S 6

Gene ID	Other Marker No.	Types	Kegg Pathway	Chromosomes	log2 (NSF5 / CNSF5)	log2 (NSF75 / CNSF75)
LOC4332360	HSP17.4/OJ1364E02.10/OSHSP17.3/OsHsp17.4/OsJ_10261	mRNA	04141///Protein processing in endoplasmic reticulum	chr3	1.33144576	1.228437478
LOC4332361	HSP18.1/OJ1364E02.11/OSHSP18.0/OsHsp18.1/OsJ_10262	mRNA	04141///Protein processing in endoplasmic reticulum	chr3	1.936771134	2.359850524
LOC4332363	HSP17.7/OJ1364E02.12/OSHSP17.7/OsJ_10263	mRNA	04141///Protein processing in endoplasmic reticulum	chr3	1.218290619	1.571030001
LOC4333036		mRNA		chr3	-1.235851657	-0.681182094
LOC4333193		mRNA		chr11	20.96965151	-21.94127078
LOC4333199		mRNA		chr11	21.54324663	-0.104706225
LOC4333877	BIP2/OsBiP2	mRNA	03060///Protein export+++04141///Protein processing in endoplasmic reticulum	chr3	1.100667524	1.081504613
LOC4334045		mRNA	03015///mRNA surveillance pathway	chr3	-1.081216584	-0.399499728
LOC4334080	HSF11/HSF4/HSFA2A/HSP2/OsHsf-11/OsJ_012039/RHSP2/rHsf4	mRNA		chr3	1.155730399	1.768965454
LOC4335189		mRNA		chr4	-1.045748864	-0.431395649
LOC4337864		mRNA		chr5	1.892372828	-0.406569959
LOC4337900		mRNA	03040///Spliceosome	chr5	-1.025310935	-0.406421328
LOC4338718	Cht-2/Cht2/OsChia1b/OsJ_18467/RC7/chitinase	mRNA	00520///Amino sugar and nucleotide sugar metabolism+++04016///MAPK signaling pathway - plant	chr5	1.402288556	0.687636789
LOC4339670		mRNA		chr5	1.893145544	-0.891975235
LOC4341225		mRNA		chr6	-1.040546897	-0.197509516
LOC4341326	HSF17/HSFA6A/OsHsf-17/OsJ_020873	mRNA		chr6	4.399748306	4.34436996
LOC4342103	P0548E04.6	mRNA		chr6	-1.080450385	-0.661837806
LOC4344618	OJ1613_G04.14	mRNA		chr8	-4.833723511	-0.323175094

Continued Table S 6

Gene ID	Other Marker No.	Types	Kegg Pathway	Chromosomes	log2 (NSF5 / CNSF5)	log2 (NSF75 / CNSF75)
LOC4345509	OJ1051_A08.22	mRNA	00310///Lysine degradation	chr8	-1.150123952	0.022254002
LOC4348651		mRNA		chr10	1.028408774	-0.070212199
LOC4349819		mRNA		chr11	-1.108701517	0.028461529
LOC4350805		mRNA		chr11	1.173629969	-0.286118769
LOC9266741		mRNA		chr4	1.231467054	-0.44946516
LOC9267065		mRNA		chr6	-1.150869938	-0.420540753
LOC9267323		mRNA		chr6	8.277818736	
LOC9267997		mRNA	04141///Protein processing in endoplasmic reticulum+++04626///Plant-pathogen interaction	chr4	1.538947106	1.742228642
LOC9268035		mRNA		chr6	1.82424252	
LOC9268124		mRNA		chr5	-1.063575525	-0.831222465
LOC9268751		mRNA		chr2	2.240961229	1.796047151

Table S 7：CNSF75 vs NSF75 KEGG Metabolic pathway-related genes

Gene ID	Other Marker No.	Types	Kegg Pathway	Chromosomes	log2 (NSF5 / CNSF5)	log2 (NSF75 / CNSF75)
LOC107275576	DJA7/DJA7B/OJ1005_D04.17/OsDjA7	mRNA		chr5	20.6090789	-21.87459296
LOC107276572	OJ1372_D12.152	mRNA		chr7	21.84273896	-23.7561104
LOC107279585		mRNA	00190///Oxidative phosphorylation	chr12	1.488743183	1.160579142
LOC4325696	HSP16.9C/OsJ_00278/Oshsp16.9C/P0443D08.3	mRNA	04141///Protein processing in endoplasmic reticulum	chr1	0.807524843	1.034076201
LOC4327469	P0005H10.6	mRNA		chr1	0.259482478	1.013777175
LOC4329612		mRNA		chr2	1.907949344	2.111178973
LOC4330496	HSP17.8/OJ1038_A06.24/OJ1311_H06.34/OsHsp17.8	mRNA	04141///Protein processing in endoplasmic reticulum	chr2	2.315648476	2.928679099
LOC4332360	HSP17.4/OJ1364E02.10/OSHSP17.3/OsHsp17.4/OsJ_10261	mRNA	04141///Protein processing in endoplasmic reticulum	chr3	1.33144576	1.228437478
LOC4332361	HSP18.1/OJ1364E02.11/OSHSP18.0/OsHsp18.1/OsJ_10262	mRNA	04141///Protein processing in endoplasmic reticulum	chr3	1.936771134	2.359850524
LOC4333193		mRNA		chr11	20.96965151	-21.94127078
LOC4334080	HSF11/HSF4/HSFA2A/HSP2/OsHsf-11/OsJ_012039/RHSP2/rHsf4	mRNA		chr3	1.155730399	1.768965454
LOC4335556		mRNA		chr4	0.336914747	1.136705557
LOC4335956	HSP23.2/OsHsp23.2/OsJ_14939	mRNA	04141///Protein processing in endoplasmic reticulum	chr4	0.888885508	1.648794062
LOC4336524		mRNA		chr4	0.887296953	1.130813293
LOC4339759	SIG5	mRNA		chr5	0.188581875	1.185065813
LOC4340201	P0681F10.2	mRNA		chr6		1.027723939
LOC4340661	HSP16.0/OsHsp16.0/OsJ_20841	mRNA	04141///Protein processing in endoplasmic reticulum	chr6	1.100149262	1.586692713
LOC4341326	HSF17/HSFA6A/OsHsf-17/OsJ_020873	mRNA		chr6	4.399748306	4.34436996
LOC4347198	OJ1123_B08.25	mRNA		chr9	0.683832917	-2.70091717

Continued Table S 7

Gene ID	Other Marker No.	Types	Kegg Pathway	Chromosomes	log2 (NSF5 / CNSF5)	log2 (NSF75 / CNSF75)
LOC4347405	HSP81-3	mRNA	04141///Protein processing in endoplasmic reticulum+++04626///Plant-pathogen interaction	chr9		-24.600932
LOC4350180	HSP21.9/OsHsp21.9	mRNA	04141///Protein processing in endoplasmic reticulum	chr11	1.069385522	2.598108085
LOC9266706		mRNA		chr10	0.620207867	-8.254731427
LOC9267997		mRNA	04141///Protein processing in endoplasmic reticulum+++04626///Plant-pathogen interaction	chr4	1.538947106	1.742228642
LOC9268751		mRNA		chr2	2.240961229	1.796047151
LOC9271813		mRNA	03010///Ribosome	chr7	9.629273388	21.99094801

Table S 8：NSF5 vs NSF75 GO：0009414 Enrichment of water metabolism-related genes

Gene ID	Other Marker No.	Types	Chromosomes	log2 (CNSF75 / CNSF5)	log2 (NSF75 / NSF5)	Qvalue (CNSF75 / CNSF5)	Qvalue (NSF75 / NSF5)
LOC4325061	OREB1/P0679C12.1	mRNA	chr1	-1.303	-1.341	7.82E-18	2.08E-20
LOC4326935	rDRP1/rab25	mRNA	chr1	0.610	0.420	1.40E-05	3.33E-04
LOC4327457		mRNA	chr1	1.520	0.877	1.42E-10	3.28E-04
LOC4327621		mRNA	chr1	-0.264	-1.063	0.150371241	5.87E-13
LOC4328073	CAT-A/OJ1442_E05.8-2/OsJ_004973/catA/catalase	mRNA	chr2	-1.355	-1.963	4.60E-08	5.46E-20
LOC4328496		mRNA	chr2	0.252	-0.618	0.353578905	0.01763523
LOC4328512	OJ1297_C09.15/OsWRKY71/wrky38	mRNA	chr2	0.546	0.424	0.001111432	0.014582635
LOC4330248	PIP1-1/PIP1.1/PIP1A/RWC-1/RWC1/aquaporin	mRNA	chr2	-0.635	-0.880	1.22E-05	2.51E-12
LOC4330265	Dhn1/Dip1/LIP9/OsDhn1/OsLIP9	mRNA	chr2	-0.739	-0.775	2.06E-05	9.05E-09
LOC4330759	OJ1288_G09.26/P0627E03.4	mRNA	chr2	-0.271	-0.625	0.195008074	2.17E-06
LOC4330838	OJ1004_A11.20	mRNA	chr2	-1.423	-1.144	1.09E-09	1.87E-08
LOC4331194	OJ1136_C04.6/OsJ_008613/PIP1-3/RWC-3/ RWC3	mRNA	chr2	-3.654	-3.584	1.02E-47	2.61E-10
LOC4331427		mRNA	chr3	3.260	2.985	2.37E-22	3.99E-19
LOC4331509	CatC/catalase	mRNA	chr3	-0.915	-0.833	1.23E-06	1.20E-04
LOC4335984	ABA1/OsABA1/OsABA2/OsZEP1/ZEP	mRNA	chr4	-0.222	-0.446	0.451298886	0.010956321
LOC4336777		mRNA	chr4	3.637	2.299	2.03E-05	0.006088195
LOC4336783	OsCDPK7	mRNA	chr4	1.218	1.423	1.28E-10	5.65E-12
LOC4336912	ACA5/OsACA5/OsACA6/OsJ_16068	mRNA	chr4	-0.705	-0.423	3.76E-05	0.00888866
LOC4338416		mRNA	chr5	0.319	0.502	0.306505006	0.002811488
LOC4339029		mRNA	chr5	3.597	3.260	0.080386983	1.53E-04
LOC4339049		mRNA	chr5	1.860	1.076	1.69E-09	1.74E-04
LOC4339797	OsJ_018965/OsPP2C53	mRNA	chr5	-1.175	-1.128	2.48E-10	1.00E-11
LOC4339892	G6PGH1/OsG6PGH1/OsJ_19864	mRNA	chr6	0.926	0.638	3.67E-05	3.80E-05
LOC4340462	P0701E03.2	mRNA	chr6	-1.006	-1.148	1.90E-07	2.38E-10
LOC4340522		mRNA	chr6	3.226	2.933	3.47E-53	2.57E-71

Continued Table S 8

Gene ID	Other Marker No.	Types	Chromosomes	log2 (CNSF75 / CNSF5)	log2 (NSF75 / NSF5)	Qvalue (CNSF75 / CNSF5)	Qvalue (NSF75 / NSF5)
LOC4341420		mRNA	chr6	1.558	1.510	2.38E-16	2.90E-14
LOC4341453	P0486H12.3	mRNA	chr6	-0.485	-0.777	0.106049357	1.04E-04
LOC4341618	P0458E02.2/P0458E02.3	mRNA	chr6	0.646	1.106	5.17E-07	4.37E-18
LOC4342410	CIPK23/OJ1027_G06.28//OsCIPK23	mRNA	chr7	0.189	0.528	0.224054385	2.14E-05
LOC4344028	DRR1/LTI6A/OJ1136_D11.102/OsJ_25274	mRNA	chr7	1.746	1.184	6.16E-09	4.66E-06
LOC4345613		mRNA	chr8	4.625	4.256	4.66E-08	2.46E-08
LOC4346391		mRNA	chr9	-4.275	-2.834	1.41E-04	1.27E-06
LOC4346972		mRNA	chr9	-0.533	-0.688	0.002840466	9.47E-07
LOC4347115	OJ1299_A11.27	mRNA	chr9	0.954	0.777	4.14E-07	6.52E-05
LOC4347566		mRNA	chr9	1.088	0.965	1.00E-10	8.89E-11
LOC4347799		mRNA	chr9	0.866	0.791	2.56E-06	4.32E-08
LOC4349248		mRNA	chr10	1.754	1.566	2.59E-13	1.04E-14
LOC4350081		mRNA	chr11	0.803	0.877	1.23E-04	2.30E-09
LOC4350451	OsJ_032468/RAB16D	mRNA	chr11	-1.600	-1.774	2.28E-19	5.03E-55
LOC4350453	OsJ_032471/RAB16B	mRNA	chr11	-0.641	-0.643	3.54E-04	5.41E-08
LOC4350454	OsJ_032472/RAB21	mRNA	chr11	0.507	0.420	0.010441494	0.004809064
LOC9270544		mRNA	chr7	0.946	1.319	0.023282283	9.45E-04
LOC9270575	OsCRY2	mRNA	chr2	-0.855	-0.514	4.87E-06	0.0013275
LOC9271913	HK3/OHK2/OsCRL2/OsHK3	mRNA	chr1	-0.756	-0.369	9.60E-10	0.01230812
LOC9272469	D3	mRNA	chr6	-2.038	-1.998	6.22E-42	6.01E-44
